# Effect of chronic intermittent hypoxia-induced HIF-1α/ATAD2 expression on lung cancer stemness

**DOI:** 10.1186/s11658-022-00345-5

**Published:** 2022-06-07

**Authors:** Shengyu Hao, Fan Li, Pan Jiang, Jian Gao

**Affiliations:** 1 Department of Pulmonary Medicine, Zhongshan Hospital, Fudan University, Shanghai, 200032 China; 2 Department of Nutrition, Zhongshan Hospital, Fudan University, 180 Fenglin Road, Shanghai, 200032 China

**Keywords:** ATAD2, Lung cancer stem cells, CIH, HIF-1α

## Abstract

**Background:**

Obstructive sleep apnea is associated with increased lung cancer incidence and mortality. Cancer stem cells (CSCs) are characterized by their self-renewing ability, which contributes to metastasis, recurrence, and drug resistance. ATPase family AAA domain-containing protein 2 (ATAD2) induces malignancy in different types of tumors. However, a correlation between ATAD2 expression and CSCs in lung cancer has not yet been reported.

**Methods:**

The relative messenger RNA (mRNA) levels of ATAD2, CD44, CD133, and hypoxia-inducible factor (HIF)-1α were determined using reverse-transcription quantitative polymerase chain reaction. ATAD2 protein levels were determined using Western blotting. Cell counting kit-8, 5-ethynyl-2′-deoxyuridine (EdU), and colony formation assays were performed to analyze the proliferation of lung cancer cells. Transwell migration and invasion assays were performed to evaluate cell migration and invasion, respectively. Tumor sphere formation analysis was used to determine tumor spheroid capacity. The link between ATAD2 and HIF-1α was verified using a dual-luciferase reporter assay. Immunofluorescence staining was performed to assess mitochondrial reactive oxygen species (mtROS) production. Flow cytometry analysis was conducted to determine the CD133 and CD44 positive cell ratio.

**Results:**

We evaluated the relative expression of ATAD2 in four lung cancer cell lines (A549, SPC-A1, H460, and H1299 cells) and found increased mRNA and protein levels of ATAD2 in lung cancer samples. ATAD2 overexpression was a poor prognostic factor for lung cancer patients. Loss of ATAD2 reduced lung cancer cell viability and proliferation. Additionally, ATAD2 knockdown repressed lung cancer cell migration, invasion, stem-cell-like properties, and mtROS production. Chronic intermittent hypoxia (CIH)-induced HIF-1α expression significantly activated ATAD2 during lung cancer progression.

**Conclusions:**

This study found that CIH induced HIF-1α expression, which acts as a transcriptional activator of ATAD2. The present study also suggests a novel mechanism by which the integrity of CIH-triggered HIF-1α/ATAD2 may determine lung cancer aggressiveness via the interplay of mtROS and stemness in lung cancer cells.

**Supplementary Information:**

The online version contains supplementary material available at 10.1186/s11658-022-00345-5.

## Introduction

Despite recent clinical breakthroughs, lung cancer is still the leading cause of cancer-related death globally. Cancer stem cells (CSCs) constitute a small proportion of tumor cells, including lung cancer cells, that exhibit growth, metastasis, and therapeutic resistance [1–3]. Various cell surface proteins, including CD133 and CD44, have been successfully identified as cancer stem cell markers [4, 5].

Obstructive sleep apnea (OSA) is defined as recurrent upper-airway collapse during sleep, leading to intermittent nocturnal hypoxia and sleep fragmentation [6]. Chronic intermittent hypoxia (CIH) is one of the main characteristics of OSA and is responsible for OSA-related diseases such as cardiovascular diseases, metabolic diseases, and various cancers. Furthermore, patients with OSA exhibit increased prevalence of cancer or cancer-related mortality for various cancers, including breast, cervical, and lung cancer [7–9]. Hypoxia plays a significant role in cancer, and hypoxia-inducible factor (HIF)-1α is an important mediator of the hypoxic response during tumor growth [10]; For instance, miR-200c inhibits migration of lung cancer cells by inhibiting HIF-1α expression [11]. Our previous study also revealed that CIH promoted lung cancer and lung CSC-like properties in an orthotopic murine model of primary lung cancer [12]. However, the increased risk associated with CIH and the effects of OSA on the natural carcinogenic process are still poorly understood and require further elucidation.

ATPase family AAA domain-containing 2 (ATAD2), a member of the AAA^+^ ATPase family [13, 14], is a promising oncoprotein that plays an essential role in tumorigenesis by regulating the proliferation, invasion, and migration of cancer cells [15–17]. Researchers have revealed the clinical and prognostic effects of ATAD2 in breast, gastric, hepatocellular, endometrial, ovarian, and lung cancer; overexpression of ATAD2 is associated with rapid mortality, poor overall survival, and disease recurrence in these cancers [18, 19]. Diverse signaling pathways of ATAD2 involved in the functions of pleiotropic oncogenes, including HIF-1α, have been verified in various human cancers; For example, Nayak et al. reported that the ATAD2 promoter binds to HIF-1α under hypoxic conditions. The clinical and prognostic value of ATAD2 have been further explored in CIH-related lung cancers.

Our findings revealed a new link between ATAD2 and lung cancer progression. We found that ATAD2 knockdown repressed lung cancer growth, metastasis, and invasion. Mechanistically, we hypothesized that CIH-induced HIF-1α activates ATAD2, which contributed to lung cancer progression by increasing the accumulation of mitochondrial reactive oxygen species (mtROS).

## Materials and methods

### Ethics statement

This study was performed in accordance with the principles of the Declaration of Helsinki. This study was approved by the Ethics Committee of Zhongshan Hospital, Fudan University [no. 2018-002(5), 8 October 2020]. All participants provided written informed consent to participate in this study.

### Patient samples and ethics approval

In the current study, Zhongshan Hospital, Fudan University provided eight pairs of lung adenocarcinoma (LUAD) tissues and matched adjacent tissues. After resection, tissues were frozen in liquid nitrogen for 30 min.

### Cell culture

A549, H460, H1299, SPC-A1, and HBE cells were obtained from the American Type Culture Collection (Manassas, VA, USA). A549, H1299, and SPC-A1 cells were grown in Roswell Park Memorial Institute 1640 medium containing 10% fetal bovine serum (FBS; Sigma-Aldrich, St. Louis, MO, USA) with 100 U/mL penicillin and 100 mg/mL streptomycin/penicillin. H460 and HBE cells were cultured in Dulbecco’s modified Eagle’s medium (DMEM; Gibco, Grand Island, NY, USA) with 10% FBS at 37 °C. The cells were cultured in a humidified atmosphere containing 5% CO_2_.

### Gene expression profiling interactive analysis (GEPIA)

GEPIA was used to analyze ATAD2 expression in patients with LUAD. We performed Student’s *t*-test to generate a *p*-value, and used 0.05 as the *p*-value cutoff.

### Kaplan–Meier plotter

We used the Kaplan–Meier plotting method to evaluate the prognostic value of ATAD2 expression in patients with lung cancer. We divided the patients into two groups with high or low ATAD2 expression based on median mRNA expression and verified using the Kaplan–Meier survival curve, the hazard ratio (HR) with 95% confidence interval (CI), and log-rank *p*-value. *p*-value < 0.05 indicates statistically significant difference.

### Cell transfection

We obtained small interfering RNA (siRNA) negative control (NC) and siRNAs targeting ATAD2 and HIF-1α from GenePharma (Shanghai, China) and performed siRNA transfection using PowerFect in vitro siRNA transfection reagent (SignaGen, Rockville, MD, USA) according to the manufacturer’s instructions. Lung cancer cells with ectopic expression of ATAD2 were transfected with ATAD2 expression plasmid purchased from GenePharma (Shanghai, China).

### Cellular proliferation assay

We conducted a proliferation assay using the BeyoClick EdU kit (Beyotime Biotechnology, Shanghai, China). Briefly, A549 and SPC-A1 cells (approximately 5 cells/wells) were seeded into a 96-well plate. After the indicated treatments, 100 μL medium containing 50 μM 5-ethynyl-2′-deoxyuridine (EdU) was added to each well. Cells were incubated for 2 h at 37 °C, fixed with 4% paraformaldehyde, and stained with Hoechst 33342 and Apollo reaction cocktail. Fluorescence microscopy (Nikon) was used to capture the merged images using Adobe Photoshop 6.0. Cell viability was assessed using a cell counting kit (CCK)-8 assay (DOJINDO, Kumamoto, Japan). Briefly, 2 × 10^3^ cells were seeded into each well of a 96-well plate. After treatment, 10 μL CCK-8 solution was added to each well and incubated at 37 °C for 1 h. Cell proliferation curves were obtained by measuring the absorbance at 450 nm at the indicated time points. These experiments were performed in triplicate.

### Colony formation assay

Cells were seeded into 6-cm dishes (500 cells/well) and exposed to the indicated treatments. After culturing for 2 weeks, colonies were fixed with 4% paraformaldehyde and stained with 0.4% crystal violet solution, and the images were captured using a camera.

### Cell migration and invasion assays

Transwell chambers with 8-µm pore size membrane inserts (BD Biosciences, San Diego, CA, USA) were used to perform cell migration and invasion assays. A549 and SPC-A1 cells were used after 48 h of transfection in this experiment. Briefly, 100 µL serum-free medium containing 1 × 10^5^ cells was added to each upper chamber. Complete medium (600 µL) was added to the lower chamber as inducer. After 48 h of incubation, we removed the cells from the upper chamber and fixed the cells in the lower chamber. After washing the chambers, cells were stained with 0.1% crystal violet and observed under an inverted microscope (Olympus Corporation, Tokyo, Japan). Five random fields of view were counted in each group. The procedures for the invasion assay were the same as those for the cell migration assay, except that the membranes were coated with 40 µL Matrigel.

### Flow cytometry analysis

After transfection with siRNA-NC or ATAD2 siRNA, SPC-A1 cells were seeded into a 12-well plate and cultured under normoxia or CIH for 48 h. At the end of the CIH cycle, cells were harvested, filtered, and centrifuged. Fluorescein isothiocyanate (FITC)-labeled anti-CD44 (555478) and APC-labeled anti-CD133 (53276) (Cell Signaling Technologies, Danvers, MA, USA) antibodies were used for surface staining. To evaluate cellular mtROS, A549 and SPC-A1 cells were treated with 5 μM MitoSOX red mitochondrial superoxide indicator (Invitrogen, Carlsbad, CA, USA) for 10 min at 37 °C and washed with phosphate-buffered saline (PBS). The data were analyzed using FlowJo software (Tree Star Inc., San Carlos, CA, USA).

### Western blotting analysis

Cells were lysed using radioimmunoprecipitation assay lysis buffer containing a protease inhibitor cocktail (P1046; Beyotime Biotechnology, Shanghai, China). The total protein concentration in the supernatant was measured (Beyotime Biotechnology, Shanghai, China). The total protein was mixed with 5× sodium dodecyl sulfate–polyacrylamide gel electrophoresis (SDS-PAGE) loading buffer and heated at 100 °C for 5 min. The proteins were separated using 10% SDS-PAGE and transferred onto a 0.45-µm-thick polyvinylidene fluoride (PVDF) membrane (EMD Millipore, MA, USA). After blocking in 5% skimmed milk at room temperature for 1 h, we incubated the PVDF membrane overnight with the primary antibody at 4 °C and horseradish peroxidase (HRP)-conjugated secondary antibody at 37 °C for 1 h. Immobilon Western Chemilum HRP substrate (EMD Millipore, Billerica, MA, USA) was used to visualize the protein bands. The primary antibodies used were anti-glyceraldehyde 3-phosphate dehydrogenase (GAPDH) (5174; Cell Signaling Technologies) and anti-ATAD2 (50563; Cell Signaling Technologies) antibodies. HRP-conjugated goat anti-rabbit immunoglobulin G (IgG) (1:20,000; Cell Signaling Technologies) or anti-mouse IgG (1:20,000; Cell Signaling Technologies) antibodies were used as secondary antibodies.

### Reverse-transcription quantitative polymerase chain reaction (RT-qPCR)

TRIzol reagent (Thermo Fisher Scientific, Waltham, MA, USA) was used to extract the total RNA from cells. A BioPhotometer Plus spectrophotometer was used to test the purity and concentration of total RNA. Total RNA was reverse-transcribed into complementary DNA (cDNA) using the ImProm-II reverse-transcription system kit (Promega, Madison, WI, USA), according to the manufacturer’s protocol. We carried out RT-qPCR with SYBR Green qPCR SuperMix (Thermo Fisher Scientific, Waltham, MA, USA) and a ABI 7500 sequence detection system (Applied Biosystems, Foster City, CA, USA). GAPDH was used as internal control for normalization of ATAD2, HIF-1α, CD133, and CD44 expression. The relative expression of the target genes was evaluated using the 2^−ΔΔCq^ method. The RT-qPCR primers used in this study are shown in Additional file 1: Table S1.

### Analysis of ATAD2 promoter activity

Briefly, A549 and SPC-A1 cells were transfected with HIF-1α-FLAG or control plasmid. After 24 h, the cells were transfected with β-galactosidase (β-gal) plasmid and wild-type or mutant ATAD2 promoter plasmid or pGL3 alkaline luciferase reporter plasmid. Luciferase activity was measured using a luciferase detection kit (Promega, Madison, WI, USA), and β-gal activity was also measured. The relative luciferase activity was measured as the ratio of Luc to β-gal activity.

### Tumor sphere formation test

After transfection, the cells were seeded in an ultralow-attachment T25 culture flask (Corning, NY, USA) for 3 weeks. DMEM/F12 serum-free medium (Invitrogen, Carlsbad, CA, USA) containing 5 μg/mL insulin, 20 ng/mL epidermal growth factor, 2% B27, and 20 ng/mL basic fibroblast growth factor was used to culture the spheres. A phase-contrast microscope (40× ; Olympus, Tokyo, Japan) was used to calculate the number of tumor spheres.

### Immunofluorescent staining

A549 and SPC-A1 cells were grown on sterilized coverslips in a six-well plate. After treatment, cells were fixed with 4% paraformaldehyde and permeabilized with 0.5% Triton X-100. The cells were then blocked with 5% bovine serum albumin (Ameresco, Solon, OH, USA) in PBS containing 0.05% Tween 20 for 1 h and incubated with 5 μM MitoSOX red mitochondrial superoxide indicator (Invitrogen, Carlsbad, CA, USA). Subsequently, the coverslips were stained with 4′,6-diamidino-2-phenylindole dihydrochloride (DAPI; 1:5000; Beyotime, Shanghai, China) and imaged using a fluorescence microscope (Nikon, Tokyo, Japan).

### Statistical analysis

GraphPad Prism 5.0 (GraphPad Software, USA) was used to analyze the data, and the results are expressed as mean ± standard deviation. One-way analysis of variance (ANOVA) with multiple comparisons using Dunnett’s test was performed to compare the differences among groups. The unpaired Student’s *t*-test was used to analyze the statistical differences between two groups. *p* < 0.05 was considered to be statistically significant.

## Results

### Overexpression of ATAD2 is associated with poor survival in LUAD patients

**Fig. 1:**
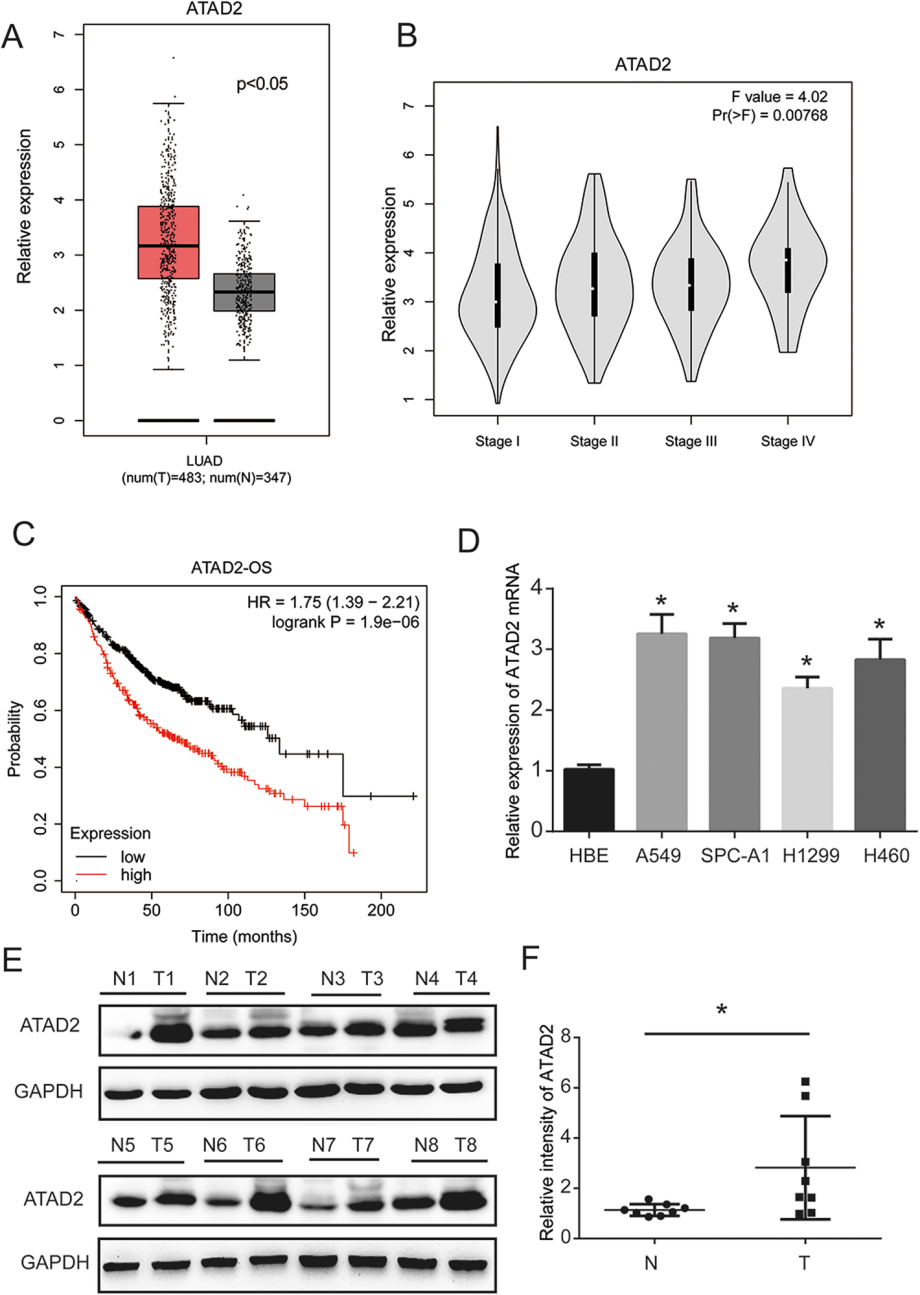
ATAD2 expression is upregulated in LUAD tissues and cells. **A** Overexpression of ATAD2 mRNA is observed in LUAD tissues compared with normal samples [gene expression profiling interactive analysis (GEPIA)]. **B** ATAD2 mRNA expression is significantly related to the cancer stage of the patient (GEPIA). **C** Prognostic feature of mRNA expression of ATAD2 in LUAD patients (Kaplan–Meier plot). Overall survival (OS) curve comparing patients with high (red) and low (black) ATAD2 expression in lung cancer plotted using the Kaplan–Meier method with a threshold of *p*-value < 0.05. **D** mRNA expression of ATAD2 is increased in lung cancer cells compared with normal lung epithelial HBE cells. **E**, **F** Protein expression of ATAD2 is increased in the tissues of lung cancer patients. *p < 0.05

The Cancer Genome Atlas (TCGA) data analysis showed that ATAD2 RNA levels were high in patients with LUAD (Fig. 1A). In addition, ATAD2 expression was significantly associated with cancer stages (Fig. 1B). As shown in Fig. 1C, high ATAD2 RNA level was related to worse overall survival outcome in LUAD, suggesting that ATAD2 may serve as a potential oncogene in lung cancer. We then determined ATAD2 expression in four lung cancer cell lines (A549, H460, H1299, and SPC-A1) and one human bronchial epithelial cell line via RT-qPCR analysis. We found that ATAD2 mRNA expression was higher in lung cancer cells than in HBE cells (Fig. 1D). Furthermore, protein expression of ATAD2 was increased in eight pairs of tissues from lung cancer patients (Fig. 1E, F). These results imply that ATAD2 is involved in lung cancer progression.

### ATAD2 affects lung cancer cell viability and proliferation

**Fig. 2:**
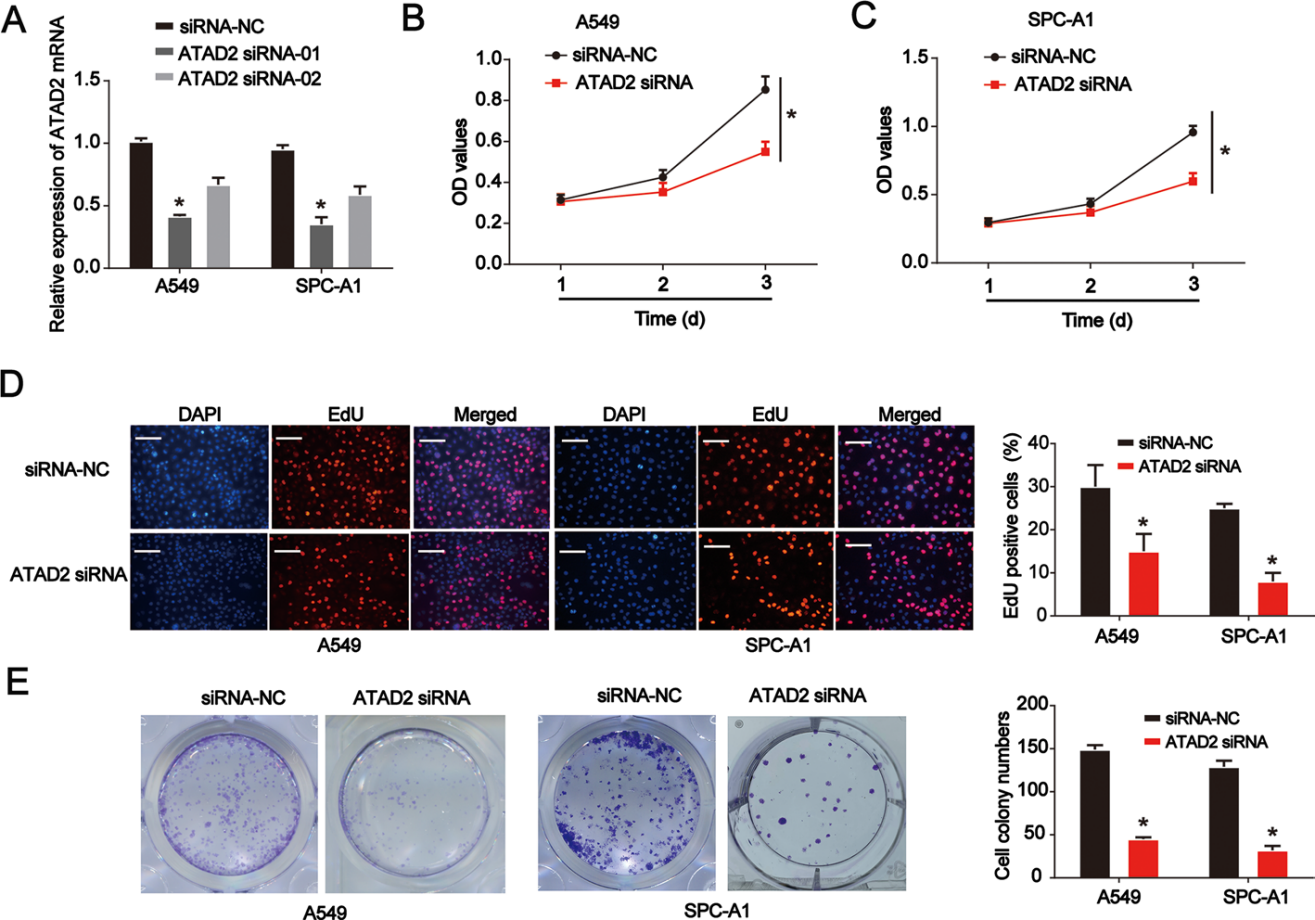
Effects of ATAD2 on lung cancer cell viability and proliferation. **A** Validation of siRNA knockdown efficiency in A549 and SPC-A1 cells as determined using RT-qPCR. **B**, **C** Cell counting kit (CCK)-8 proliferation assay in siRNA-negative control (NC) or siRNA-ATAD2-transfected A549 and SPC-A1 cells. **D** 5-Ethynyl-2′-deoxyuridine (EdU) proliferation assay using siRNA-NC- or siRNA-ATAD2-transfected A549 and SPC-A1 cells. Scale bar = 100 μm. **E** Colony-formation proliferation assay in siRNA-NC- or siRNA-ATAD2-transfected A549 and SPC-A1 cells. Error bars indicate mean ± standard deviation (SD). All experiments performed in triplicate. *p < 0.05

Next, we explored the effects of ATAD2 on lung cancer tumorigenesis. A549 and SPC-A1 cells were used in subsequent experiments. After transfection with the designed siRNA for 48 h, ATAD2 expression was determined in both cell lines. As indicated in Fig. 2A, we selected siRNA-01 to improve the knockdown efficiency. By performing a CCK-8 assay, we observed that ATAD2 knockdown repressed lung cancer cell viability (Fig. 2B, C). A similar growth-inhibiting effect was consistently verified using the EdU assay in both cell lines (Fig. 2D). Furthermore, knockdown of ATAD2 reduced the colony-forming ability of both cell types (Fig. 2E). These results suggest that ATAD2 is essential for growth of lung cancer cells.

### Effects of ATAD2 knockdown on lung cancer cell migration, invasion, and stem-cell-like properties

**Fig. 3:**
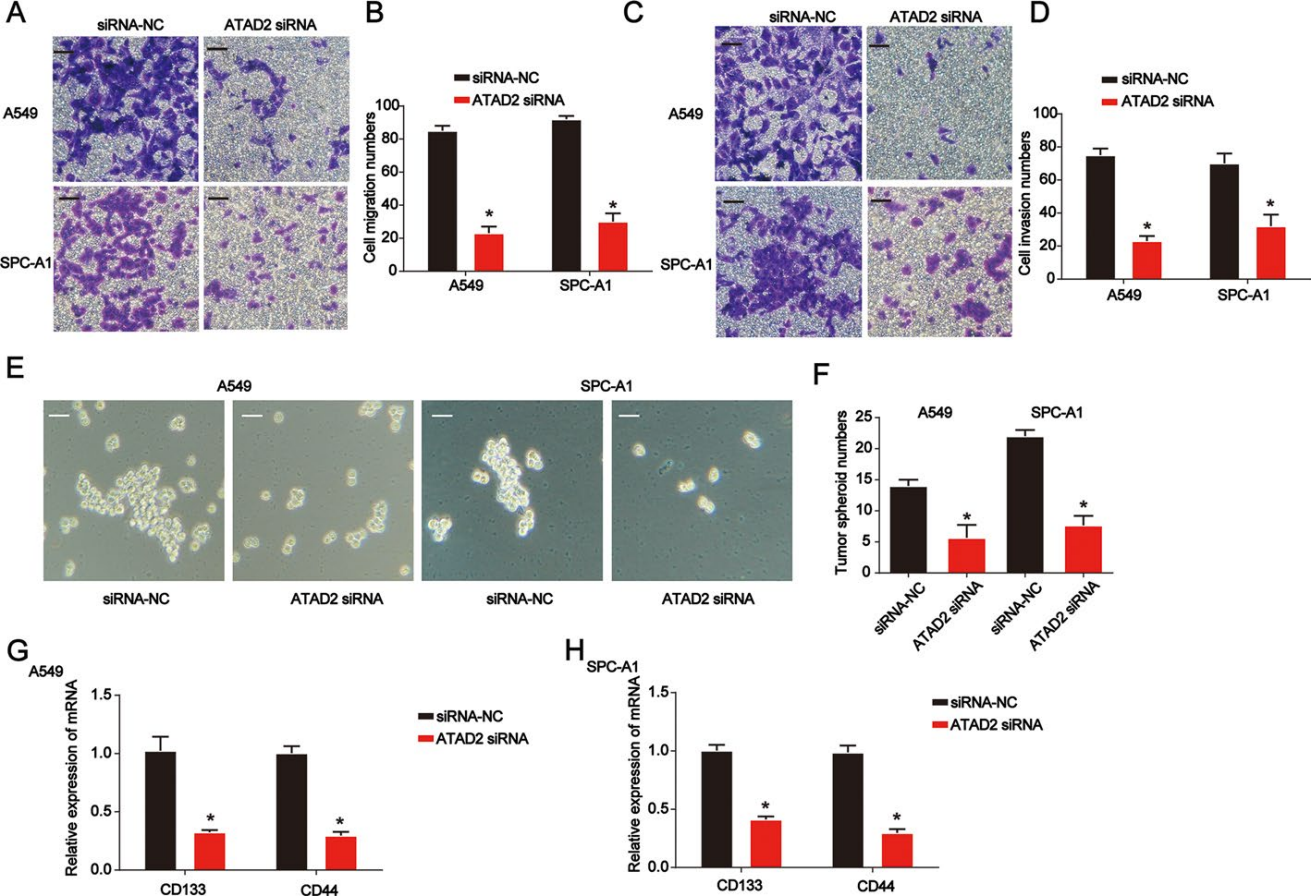
Effects of ATAD2 knockdown on lung cancer cell migration, invasion, and stemness. **A**, **B** Transwell migration assay using siRNA-NC- or siRNA-ATAD2-transfected A549 and SPC-A1 cells. **C**, **D** Transwell invasion assay using siRNA-NC- or siRNA-ATAD2-transfected A549 and SPC-A1 cells. **E**, **F** Tumor sphere formation assay using siRNA-NC- or siRNA-ATAD2-transfected A549 and SPC-A1 cells. Scale bar = 100 μm. **G**, **H** CD133 and CD44 mRNA levels in siRNA-NC- or siRNA-ATAD2-transfected A549 and SPC-A1 cells. Error bars indicate mean ± SD. All experiments performed in triplicate. *p < 0.05

Migration and invasion are the main characteristics and life-threatening aspects of lung cancer. Hence, we examined the effects of ATAD2 knockdown on the migration and invasion of A549 and SPC-A1 cells. ATAD2 knockdown suppressed cell migration (Fig. 3A, B). In addition, we observed decreased cell invasion ability after ATAD2 siRNA treatment using the Transwell invasion test (Fig. 3C, D). As shown in Fig. 3E, F, tumor sphere growth was significantly repressed by knockdown of ATAD2. In addition, CD133 and CD44 mRNA expression was significantly decreased by loss of ATAD2 in lung cancer cells (Fig. 3G, H). These results indicate that loss of ATAD2 represses lung cancer cell migration and invasion.

### Effects of ATAD2 overexpression on mtROS production in lung cancer cells

**Fig. 4:**
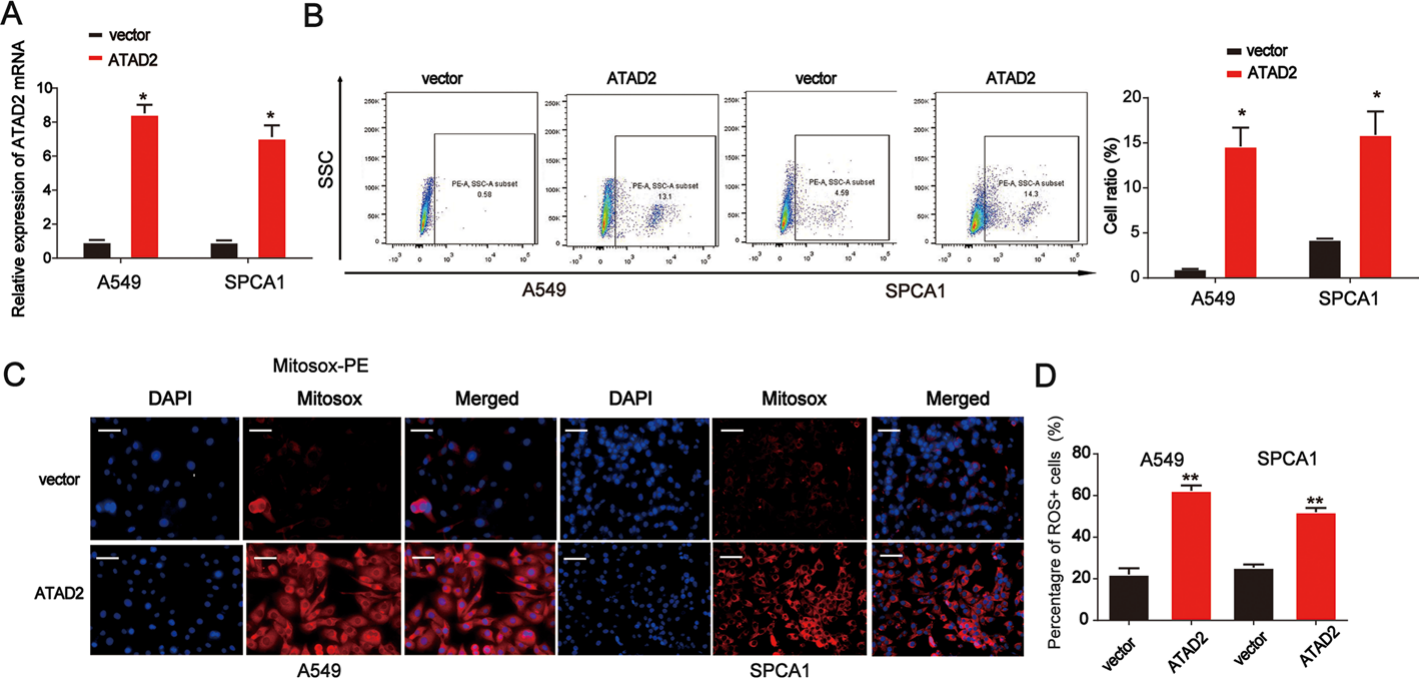
Effect of decreased ATAD2 levels on mtROS production in lung cancer cells.** A** ATAD2 mRNA expression in lung cancer cells transfected with the ATAD2 overexpression plasmid. **B** Cellular mtROS accumulation in A549 and SPC-A1 cells assessed using flow cytometry analysis. **C**, **D** Nuclei are stained with Hoechst (blue), and mitochondria ROS are stained using MitoSOX-red in A549 and SPCA1 cells. Merged panels demonstrate the number of MitoSOX-red positive cells among the total cells. Scale bar = 100 μm. Error bars indicate mean ± SD. All experiments performed in triplicate. *p < 0.05

ROS are essential regulators of CSCs, and mtROS are a primary source of ROS. As shown in Fig. 4A, ATAD2 expression was significantly increased in lung cancer cells. We found that ATAD2 overexpression notably triggered mtROS production in both cell lines, as determined using flow cytometry and immunofluorescence staining (Fig. 4B–D). These results indicate that ATAD2 is involved in mtROS production in lung cancer cells.

### CIH-induced HIF-1α activates ATAD2 during lung cancer progression

**Fig. 5:**
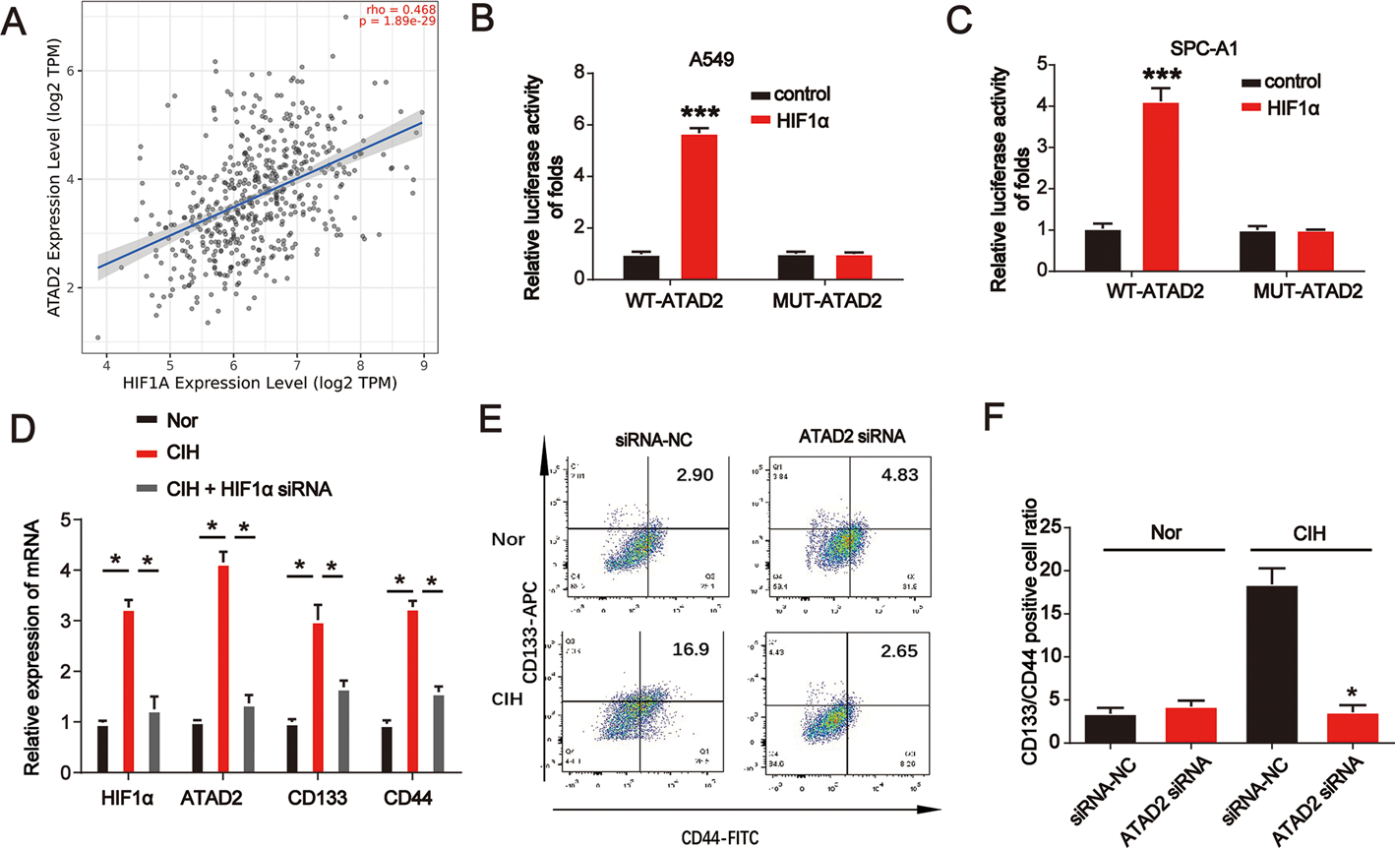
CIH-induced HIF-1α activated ATAD2 during lung cancer progression. **A** Gene correlation between ATAD2 and HIF-1α in LUAD determined by consulting http://timer.comp-genomics.org/. **B**, **C** Analysis of ATAD2 promoter activity. Cells were transfected with the HIF-1α-FLAG or control plasmid for 24 h. A549 and SPC-A1 cells were transfected with an ATAD2 promoter plasmid or a plasmid containing a mutated ATAD2 promoter sequence for another 24 h. **D** HIF-1α, ATAD2, CD133, and CD44 mRNA expression levels in A549 cells incubated under normoxia, CIH, or CIH combined with HIF-1α. **E**, **F** Flow cytometry analysis of A549 cells transfected with siRNA-NC or siRNA-ATAD2 under normoxia or CIH. Error bars indicate mean ± SD. All experiments performed in triplicate. *p < 0.05

As shown in Fig. 5A, there is a positive link between ATAD2 and HIF-1α in LUAD as determined after consulting http://timer.comp-genomics.org/. Furthermore, overexpression of HIF-1α significantly enhanced the luciferase activity of the ATAD2 promoter but did not alter the luciferase activity of the CD44 promoter when the HIF-1α binding site was mutated in A549 and SPC-A1 cells (Fig. 5B, C). These results imply that HIF-1α acts as a transcriptional promoter of ATAD2. As shown in Fig. 5D, CIH significantly induced HIF-1α, ATAD2, CD133, and CD44 mRNA expression in A549 cells, which was reduced by the siRNA of HIF-1α. Furthermore, flow cytometry analysis revealed that ATAD2 siRNA repressed the CD133 and CD44 positive cell ratio induced by CIH (Fig. 5E, F). These results suggest that CIH-induced HIF-1α activates ATAD2 during lung cancer progression.

## Discussion

OSA is considered to be an important promoter of the occurrence and development of lung cancer [20]. In this study, we aimed to evaluate the association between OSA and lung cancer. CIH can enhance apoptosis resistance and metastasis of lung cancer cells by increasing expression of HIF-1α [21]. In addition, CIH can exacerbate lung cancer progression in mouse models [22].

In this study, our data show that an increase in ATAD2 expression in lung cancer cells and tissues indicates poor prognosis for lung cancer patients. Knockdown of ATAD2 reduced A549 and SPC-A1 cell proliferation, migration, and invasion. Intermittent hypoxia caused an increase in HIF-1α levels and promoted ATAD2 expression and mtROS production, resulting in CSC-like characteristics, which may partly explain the promotion of lung cancer progression by OSA. Our research reveals the possible mechanism underlying the involvement of ATAD2 in advancing CIH-induced lung cancer. To the best of the authors’ knowledge, these findings support the biological hypothesis of adverse outcomes of OSA in lung cancer.

ATAD2 contains a bromodomain and an ATPase domain, which maps to chromosome 8q24 in cancer [23]. The structure of ATAD2 suggests that its functions are related to genome regulation, such as cell proliferation, differentiation, and apoptosis. ATAD2 is a member of the AAA^+^ ATPase family, and its overexpression in cancer tissue indicates poor prognosis [15, 24]. An increase in ATAD2 expression has been identified in many tumors, including hepatocellular, breast, and lung cancer [25]. ATAD2 is also required for prostate cancer cell growth [26].

Previous studies have found that high ATAD2 expression in patients with lung cancer suggests poor prognosis, which is consistent with our findings. In this study, increased expression of ATAD2 was negatively correlated with the overall survival rate of lung cancer patients. In addition, upregulation of ATAD2 promoted lung CSC characteristics. In contrast, downregulation of ATAD2 inhibits CSC properties in esophageal squamous cell carcinoma by blocking the Hedgehog signaling pathway [27]. Furthermore, we found that ATAD2 overexpression significantly induced mtROS production in A549 and SPC-A1 cells. Therefore, we hypothesized that ATAD2 overexpression induces stemness of lung cancer cells via mtROS production.

HIF-1 is a crucial oncogenic transcription factor in hypoxic environments [28, 29] and has two subunits: HIF-1α and HIF-1β. Under hypoxic conditions, HIF-1α accumulates and transfers to the nucleus and combines with HIF-1β to form activated HIF-1 [30, 31], which regulates the target gene expression. These genes are involved in cell proliferation, migration, invasion, and metabolism [32]. Abnormal activation of HIFs enables some cancer cells to obtain “stemness” abilities [33]. HIF-1 and HIF-2 transcription factors share many target genes, promoting CSC formation and amplification in cancer [34–36]. CIH occurring during sleep apnea activates HIF-1 during myocardial injury [37]. Moreover, HIF-1α-dependent upregulation of ATAD2 promotes proliferation and migration of stomach cancer cells [38]. Lysyl oxidase-like 2 (LOXL2) overexpression or hypoxia affects hepatocellular carcinoma progression by promoting ATAD2 expression [39]. Hypoxic stabilization of mRNA is independent of HIF but requires mtROS production [40]. Our study observed that the CIH-induced tumor microenvironment with upregulated HIF-1α and ATAD2 played a critical role in lung cancer progression and stemness. HIF-1α-activated ATAD2 contributed to lung cancer progression by increasing mtROS levels.

However, our study has some limitations. First, the sample size of patients with lung cancer was relatively small; therefore, more patients with LUAD suffering from CIH should be collected for further analysis. Second, only in vitro experiments were performed, whereas no in vivo animal experiments could be performed in this study. Third, the screening process for the upstream and downstream molecules of ATAD2 was not fully solid.

In conclusion, the results above suggest that ATAD2 expression was significantly upregulated in lung cancer tissues and significantly associated with the clinical stage of lung cancer. Additionally, ATAD2 overexpression may serve as a poor prognostic factor for lung cancer patients. Furthermore, we revealed that CIH-induced HIF-1α activates ATAD2 expression, contributing to lung cancer progression by increasing mtROS levels.

## Supplementary Information

**Additional file 1: Table S1. **Primer sequence for RT‐qPCR.

## Data Availability

The data that support the findings in this study are available upon reasonable request.

## Abbreviations

OSA: Obstructive sleep apnea
CSCs: Cancer stem cells
CIH: Chronic intermittent hypoxia
mtROS: Mitochondrial ROS
Nor: Normoxia
ATAD2: ATPase family AAA domain-containing protein 2
HIF-1α: Hypoxia-inducible factor 1 alpha
qRT-PCR: Real-time quantitative reverse-transcription polymerase chain reaction
