# Supplementary material for: Effect of chronic intermittent hypoxia-induced HIF-1α/ATAD2 expression on lung cancer stemness
Source: Cell Mol Biol Lett. 2022 Jun 7;27:44. doi: 10.1186/s11658-022-00345-5 (PMC9172155; doi:10.1186/s11658-022-00345-5)
Supplement: Supplementary file 1 — Additional file 1: Table S1. Primer sequence for RT‐qPCR. [file 11658_2022_345_MOESM1_ESM.docx]

**Additional file 1:** **Table S1. Primer sequence for RT‐qPCR.**

| Genes | Forward (5’-3’) | Reverse (5’-3’) |
| --- | --- | --- |
| GAPDH  β-actin | GCACCGTCAAGGCTGAGAAC  CACCATTGGCAATGAGCGGTTC | TGGTGAAGACGCCAGTGGA  AGGTCTTTGCGGATGTCCACGT |
| CD44  CD133  HIF1α  ATAD2 | GCATTGCAGTCAACAGTCGAAGA  CCTCCCTGTTGGTGATTTGTAT  GCCAGATCTCGGCGAAGTAA  GGAATCCCAAACCACTGGACA | CCTTGTTCACCAAATGCACCA  CCTTGTCCTTGGTAGTGTTGT CAAATCACCAGCATCCAGAAGT  GGTAGCGTCGTCGTAAAGCACA |
